# Effect of Ultrasound Treatment on the Structural and Techno-Functional Properties of Feather-Derived Keratin

**DOI:** 10.3390/molecules31214037

**Published:** 2026-11-09

**Authors:** Nisa Akkuzu, Betül Unur, Rukiye Gundogan, Tuncay Özdemir, Gulsah Karabulut, Ebru Firatligil, Asli Can Karaca

**Affiliations:** 1 Department of Food Engineering, Faculty of Chemical and Metallurgical Engineering, Istanbul Technical University, 34469 Maslak, Istanbul, Türkiye; nisakkuzu@itu.edu.tr (N.A.); unur24@itu.edu.tr (B.U.); gundogan17@itu.edu.tr (R.G.); ebruf@itu.edu.tr (E.F.); 2 Senpilic Gida San. A.S., 54700 Geyve, Sakarya, Türkiye; tuncay.ozdemir@senpilic.com.tr; 3 Department of Food Engineering, Faculty of Engineering, Sakarya University, 54187 Serdivan, Sakarya, Türkiye; gkarabulut@sakarya.edu.tr

**Keywords:** keratin, ultrasound, techno-functional properties, protein structure, antioxidant activity, emulsifying and foaming properties

## Abstract

Chicken feathers, an underutilized poultry-processing by-product, are a rich keratin source whose native disulfide-crosslinked structure limits solubility and functional applicability. This study investigated the effects of probe ultrasound treatment (30–60% amplitude, 10 min) on the structural and techno-functional properties of alkali-extracted chicken feather keratin (CFK) to support its valorization in various applications. Control (untreated) and ultrasound-treated CFK samples were characterized by FTIR, SDS–PAGE, free sulfhydryl (–SH) content, ζ-potential, surface hydrophobicity, antioxidant activity (ABTS and CUPRAC assays), in vitro protein digestibility, water solubility, oil-holding capacity, and emulsifying and foaming properties. FTIR and SDS–PAGE revealed that the characteristic amide bands and apparent molecular-weight profile of keratin were mainly preserved following ultrasound treatment, whereas Amide I deconvolution indicated an amplitude-dependent reduction in native β-sheet content and Ellman’s assay showed a loss of free –SH groups. Ultrasound treatment enhanced several functional properties in an amplitude-dependent manner. Surface hydrophobicity, oil-holding capacity, emulsifying activity, and ABTS-based antioxidant activity showed the highest values at 40% amplitude, while water solubility and emulsion stability were highest at 60%. The in vitro digestibility remained above 90% for all samples. These findings show that ultrasound treatment can modify the functional performance of feather keratin in an amplitude-dependent manner without marked changes in its apparent molecular-weight profile or its major amide bands, supporting its potential as a functional ingredient for various applications.

## 1. Introduction

The global poultry industry generates vast quantities of feather waste as an inevitable by-product of processing; in 2020, approximately 100.5 million tons of chicken meat production yielded more than 4.7 million tons of chicken feathers worldwide [1]. Feathers are composed of up to 90% keratin, a structural protein with considerable potential as a sustainable, low-cost biopolymer source [2]. Yet, most feather waste is still landfilled, incinerated, or converted into low-value feather meal [1], representing a considerable loss of a protein resource that could be valorized into higher-value food, feed, or industrial ingredients.

Unlike globular proteins, feather (β-)keratin has a highly cross-linked and stable structure maintained by disulfide bonds, hydrogen bonds, and hydrophobic interactions, which contributes to its rigidity and resistance to chemical, physical, and enzymatic degradation [3,4]. Extracting soluble keratin from this matrix therefore requires treatments that disrupt its highly stable structure, most commonly alkaline or reductive hydrolysis. Enzymatic methods have also been explored as an alternative to improve extraction yield while preserving peptide integrity [5,6,7].

Once extracted, keratin and its hydrolysates show functional properties, including solubility, oil-holding capacity, emulsifying and foaming properties, and antioxidant activity, that are relevant to a wide range of applications [8]. Water-soluble keratin fractions are already recognized under the International Nomenclature Cosmetic Ingredient (INCI) name “Hydrolyzed Keratin” and used as natural emulsifying, moisturizing, and conditioning agents in cosmetic and personal-care formulations [9]. However, these functional properties depend on the extent and type of structural changes induced during processing, and the choice of extraction and modification method can therefore influence the final performance of the keratin.

Ultrasound treatment has emerged as an attractive physical processing tool for modifying protein structure and functionality via acoustic cavitation, which can promote localized generation of free radicals capable of cleaving and oxidizing protein disulfide bonds [10]. In feather keratin, ultrasound has been applied mainly as an extraction aid. For instance, Qin et al. [11] used ultrasound to enhance chicken feather disintegration and keratin solubility during hydrolysis. More recently, Perdana et al. [12] combined ultrasonication with ionic-liquid extraction of duck feather keratin. Furthermore, Raydan et al. [13] investigated ultrasound-assisted alkaline hydrolysis at different temperatures to reveal the unfolding and refolding pathways of duck feather keratin. In addition to keratin, ultrasound has also been shown to modify the structural, functional, and antioxidant properties of other food proteins, such as soy protein isolate [14], chicken plasma protein [15], and shiitake mushroom protein [16] in an amplitude-dependent manner.

While previous studies demonstrate that ultrasound can affect keratin extraction efficiency and structure, ultrasound has generally been applied as part of the extraction process; thus its effect cannot be separated from that of the extraction conditions. To the best of our knowledge, the use of ultrasound as a separate post-extraction treatment of alkali-extracted keratin, compared with an untreated control prepared under identical extraction conditions, has not been reported. Moreover, previous studies have examined a limited set of structural or functional properties, such that the relationship between ultrasound amplitude and the wider structural and techno-functional profile of feather keratin, including secondary structure, free–SH content, surface charge, hydrophobicity, antioxidant capacity, digestibility, solubility, oil-holding capacity, emulsifying and foaming properties, remains poorly established. To address this gap, the present study systematically investigates the effect of probe ultrasound treatment, applied over a range of amplitudes (30–60%) to alkali-extracted chicken feather keratin, on its structural characteristics and techno-functional properties. This work aims to investigate how ultrasound amplitude affects the structural and techno-functional properties of feather keratin and to identify treatment conditions that may support its valorization as an underutilized poultry by-product for value-added applications.

## 2. Results and Discussion

### 2.1. Crude Protein Content, Extraction Yield, and Protein Recovery

The crude protein content, extraction yield, and protein recovery of the chicken feathers and the alkali-extracted keratin (CFK) are summarized in Table 1. The raw feathers contained 87.06 ± 1.24% crude protein, confirming their potential as a protein-rich raw material for keratin extraction. Alkaline extraction followed by isoelectric precipitation at pH 4.4 gave an extraction yield of 16.87 ± 0.21% (dry basis), and the recovered CFK contained 74.38 ± 0.62% crude protein. Based on these values, 14.35 ± 0.49% of the protein in the feathers was recovered in the freeze-dried CFK. The extraction yield (16.87 ± 0.21%) is within the range reported by Souza et al. [6] for NaOH extraction of keratin from feather meal followed by isoelectric precipitation at pH 4.4 (8.3–20.0 wt.%, expressed as dried keratin per initial mass), where the highest value was obtained with 2 mol/L NaOH at 15 °C for 90 min. Those authors also observed lower yields at higher extraction temperatures and noted that methods using reducing agents give higher yields, but require toxic chemicals [6]. Mengistu et al. [5] reported a protein yield of 89.71% under optimized conditions (0.65 N NaOH, 70 °C, 1 h). However, that value was calculated from Biuret measurements of the hydrolysate, not from the mass of dried, precipitated keratin, so it cannot be compared directly with our yield. The crude protein content of the recovered CFK (74.38 ± 0.62%) shows that about one quarter of the freeze-dried material was not protein. This fraction was not characterized in the present study and may include moisture, salts formed during neutralization, and residual lipids or other non-protein compounds. In addition, the Kjeldahl method measures all nitrogen, including that of peptides and free amino acids, therefore the crude protein values do not distinguish intact keratin from smaller peptides. The protein recovery of 14.35% suggests that a large part of the feather protein was either not solubilized or remained in the supernatant after precipitation at pH 4.4. Ash content, residual alkali, and the degree of hydrolysis were not determined in the present study. The SDS–PAGE profile shows that the extracted keratin is a mixture of polypeptides, and the extent of peptide-bond cleavage during extraction was not measured. Therefore, the control should be regarded as alkali-extracted keratin that may already be partly hydrolyzed, not as intact keratin. Alkaline hydrolysis can produce small peptides that do not precipitate at the isoelectric pH, and lower yields at higher temperature have been reported [6]. Lower yields under harsher alkaline conditions have been attributed to peptide degradation [5,13]. The protein remaining in the supernatant was not measured.

### 2.2. Fourier Transform Infrared Spectroscopy

**Figure 1:**
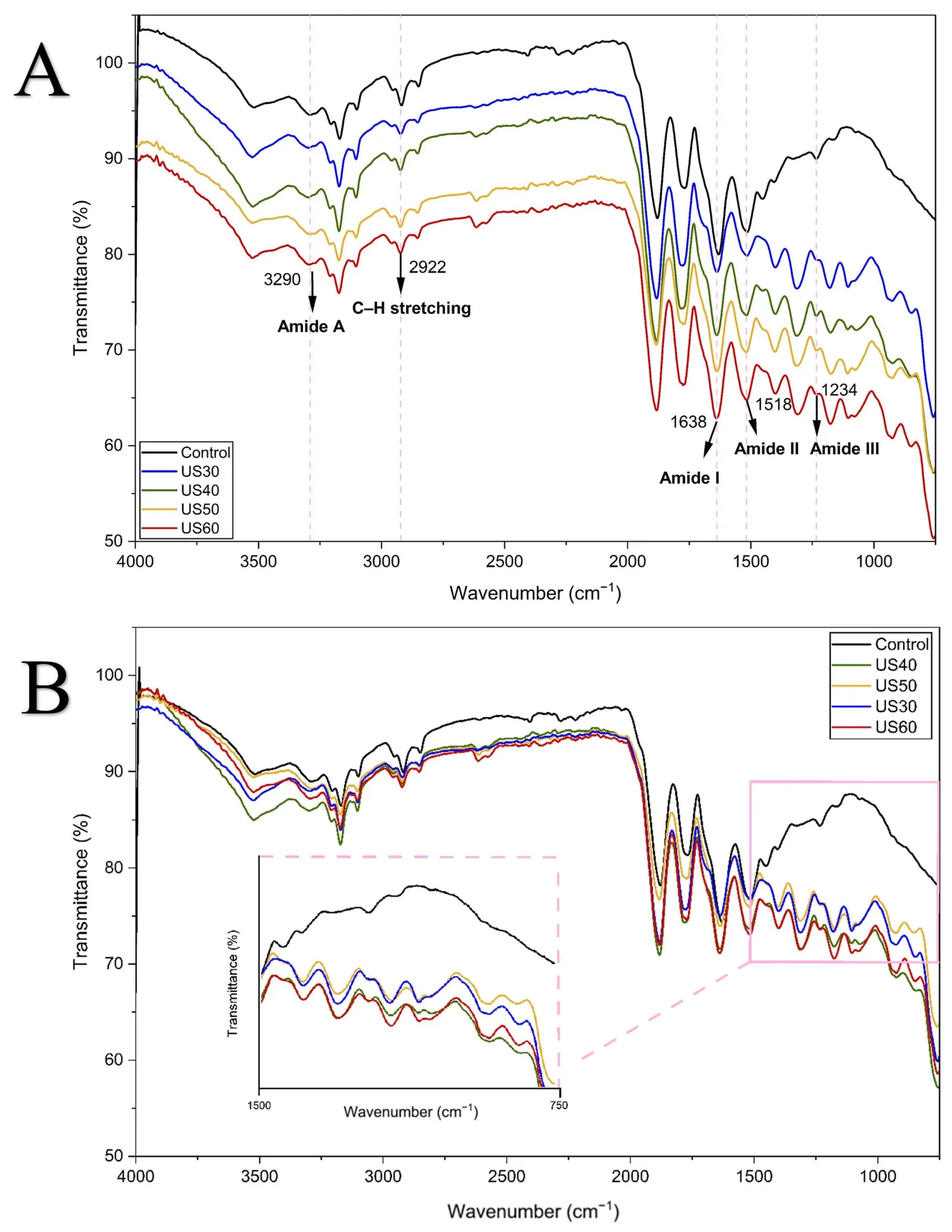
(**A**,**B**) FTIR spectra of untreated and ultrasound-treated chicken feather keratin (CFK) samples: (**A**) FTIR spectra of CFK samples; (**B**) FTIR spectra of the same samples without vertical offset, with an inset showing an enlarged view of the 1500–750 cm^−1^ region. Control represents untreated alkali-extracted CFK, while US30, US40, US50, and US60 represent CFK samples treated with ultrasound at amplitudes of 30, 40, 50, and 60%, respectively.

The FTIR spectra of untreated and ultrasound-treated chicken feather keratin (CFK) samples are presented in Figure 1. All samples, regardless of treatment, exhibited the characteristic absorption bands of keratin: Amide A at 3290 cm^−1^, corresponding to N–H stretching; a band at 2922 cm^−1^ assigned to C–H stretching; Amide I at 1638 cm^−1^, attributed to C=O stretching; Amide II at 1518 cm^−1^, associated with N–H bending coupled with C–N stretching; and Amide III at 1234 cm^−1^, related to C–N stretching and N–H bending [11]. These band positions are characteristic of keratin. The secondary structure composition is evaluated by Amide I deconvolution below. The observed bands are also consistent with the values reported for chicken feather keratin [7] (Amide A 3275, Amide I 1634, Amide II 1517, Amide III 1239 cm^−1^) and for ultrasound-extracted duck feather keratin [12] (~3280 and 1630 cm^−1^ for the O–H/N–H and Amide I bands, respectively).

The positions of these five major bands did not shift between the untreated control and the US30–US60 samples, indicating that the characteristic amide bands of keratin were retained after ultrasound treatment at 30–60% amplitude. A similar finding was reported by Qin et al. [11], who found that ultrasound treatment of chicken feather keratin did not change the characteristic chemical groups or produce new functional groups until 4 h of treatment.

However, below the Amide III region, an ultrasound-dependent effect was observed in the skeletal region of the spectra (750–1200 cm^−1^) (Figure 1B). The control spectrum showed only weak, poorly resolved absorption in this range, whereas the US30–US60 spectra displayed additional bands at approximately 856, 934, 1066, and 1153 cm^−1^. These bands were not observed in the control sample, appeared at the lowest amplitude tested (US30), and showed a slight increase in relative intensity at higher amplitudes. The bands at approximately 1066 and 1153 cm^−1^ are close to those previously assigned to cystine-S-monoxide (1075–1080 cm^−1^) and cystine-S-dioxide (1137 cm^−1^) in wool and hair keratin [17,18], although the positions differ by about 10–16 cm^−1^, and the assignment is therefore tentative. All samples, including the control, were dispersed in the same PBS and freeze-dried; therefore, residual phosphate would be expected in all spectra; the bands were absent in the control, but a contribution of phosphate, or of ultrasound-induced changes in the dried salt–protein matrix, cannot be completely excluded. Ultrasonic cavitation can generate reactive species, including free radicals, and the extent of these effects depends on the acoustic power applied [10]. The decrease in free –SH content after ultrasound treatment (Section 2.3) is consistent with the oxidation of sulfur-containing residues, but neither measurement identifies the products formed. The origin of the bands at 856 and 934 cm^−1^ was not determined. No distinct S–H stretching band was resolved in the 2550–2600 cm^−1^ region in any spectrum; only a very weak feature at about 2610–2620 cm^−1^ was observed in the ultrasound-treated samples and was not assigned.

The absence of a significant shift in the Amide I peak position does not necessarily indicate that the secondary structure was not altered. Although the main Amide I peak position remained stable, deconvolution and curve-fitting analysis (1600–1700 cm^−1^, Figures S1–S5) revealed slight conformational changes across treatments (Table 2). The secondary structure was composed of α-helices (55.54–57.16%), followed by native β-sheets (25.42–28.52%), β-turns (11.20–12.13%), and intermolecular β-sheets (4.54–6.02%). While ultrasound treatment did not significantly alter α-helix or β-turn contents (*p* > 0.05), it induced a significant decrease in native β-sheets from 28.52% in untreated keratin to 27.05% (US40), 25.42% (US50) and 26.27% (US60) (*p* < 0.05). This reduction in native β-sheet content indicates a partial change in secondary structure, which may result from the disruption of local inter-residue interactions by acoustic cavitation [12,15]. Similar conformational reorganizations induced by ultrasound-assisted processing have been highlighted by Raydan et al. [13], who reported alterations in the relative proportions of β-sheets and disordered structures even when the overall Amide I peak position remains stable. Overall, the FTIR spectra and quantitative deconvolution results indicate that while the positions of the major amide bands remained unchanged and the SDS–PAGE band profiles were similar (Section 2.4), ultrasound treatment was accompanied by an amplitude-dependent partial unfolding of native β-sheets and a loss of free –SH groups.

### 2.3. Free Sulfhydryl (–SH) Content

**Figure 2:**
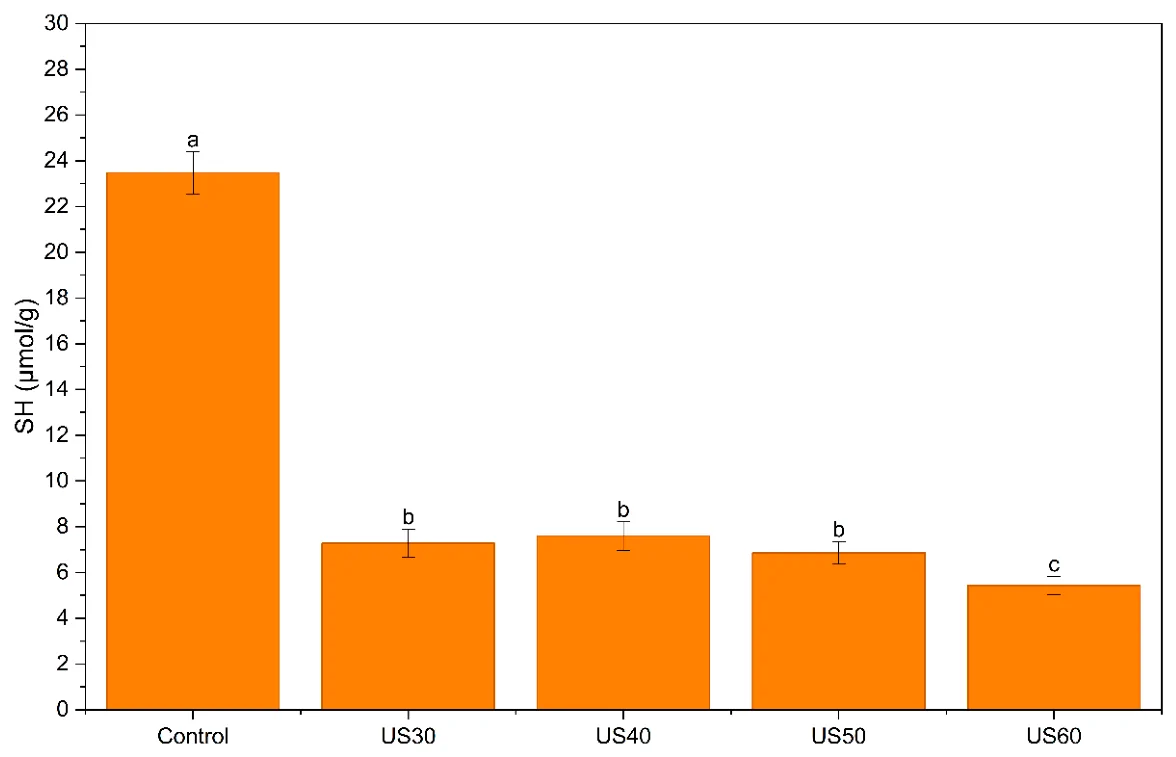
Effect of ultrasound treatment at different amplitudes (30, 40, 50, and 60%) on the free sulfhydryl (–SH) group content of chicken feather keratin (CFK). Different lowercase letters indicate statistically significant differences among samples (*p* < 0.05).

Free –SH content was determined to examine possible changes in the sulfur-containing residues of keratin after sonication, as suggested by the new bands observed in the FTIR spectra (Section 2.2). The control contained 23.5 µmol/g of free –SH (Figure 2). Ultrasound reduced this value at all amplitudes. The US30, US40, and US50 samples contained 6.9–7.6 µmol/g and did not differ significantly (*p* > 0.05), while the US60 sample had the lowest content (5.4 µmol/g; *p* < 0.05). This corresponds to a loss of about 68–71% at 30–50% amplitude and 77% at 60% amplitude.

Previous studies reported different effects of ultrasound on the –SH content of proteins, ranging from a decrease, attributed to thiol oxidation, to an increase, attributed to the exposure of buried thiol groups or the cleavage of disulfide bonds [19,20,21,22]. In feather keratin treated with a deep eutectic solvent, disulfide cleavage was reported to result in an increase in free –SH (from 1.3 to 3.0 nmol/mg), although the values stayed below that of native feathers (11.7 nmol/mg), a difference attributed to the destruction of cysteine residues and further reactions of –SH groups [23]. Decreases in free or total –SH after sonication have also been reported for beef myofibrillar protein, where the loss was greater at the higher power and was linked to the oxidation of –SH groups to disulfide bonds by radicals formed from water [19]. Similar decreases were found in faba bean protein (23–28%) [20] and milk protein concentrate (33–38%) [21]. Similarly, a lower cysteine content, with unchanged cystine, was reported in sonicated feather keratin by Qin et al. [11], who concluded that cysteine is more easily degraded under ultrasound. The larger loss at 60% amplitude in the present study agrees with the power dependence reported by Amiri et al. [19].

An increase in free –SH after sonication was explained by the unfolding of proteins and exposure of buried thiols, which offset thiol oxidation in raw milk [21]. In oxidized soy protein aggregates, free –SH increased at medium power through disulfide cleavage and decreased again at 600 W [22]. The net effect therefore depends on the protein, the matrix, and the intensity, and on whether buried or only accessible thiols are measured. In the present study, mainly accessible –SH groups were measured. Unfolding would be expected to increase this fraction, so the observed decrease suggests that thiol loss outweighed exposure.

The decrease in free –SH is consistent with the FTIR bands at about 1066 and 1153 cm^−1^, which are close to those assigned to oxidized cystine [17,18]. However, both results are indirect. A lower Ellman value may result from the oxidation of thiols to disulfides or to higher oxidation states, or from thiols becoming less accessible in aggregates, and the assay cannot distinguish between these. Total –SH and disulfide contents were not measured, and SDS–PAGE was run under reducing conditions, so intermolecular disulfide bonds could not be detected. Therefore, the results indicate a loss of free thiols, most probably by oxidation, but further studies are needed to confirm this mechanism.

### 2.4. Molecular Weight Distribution

**Figure 3:**
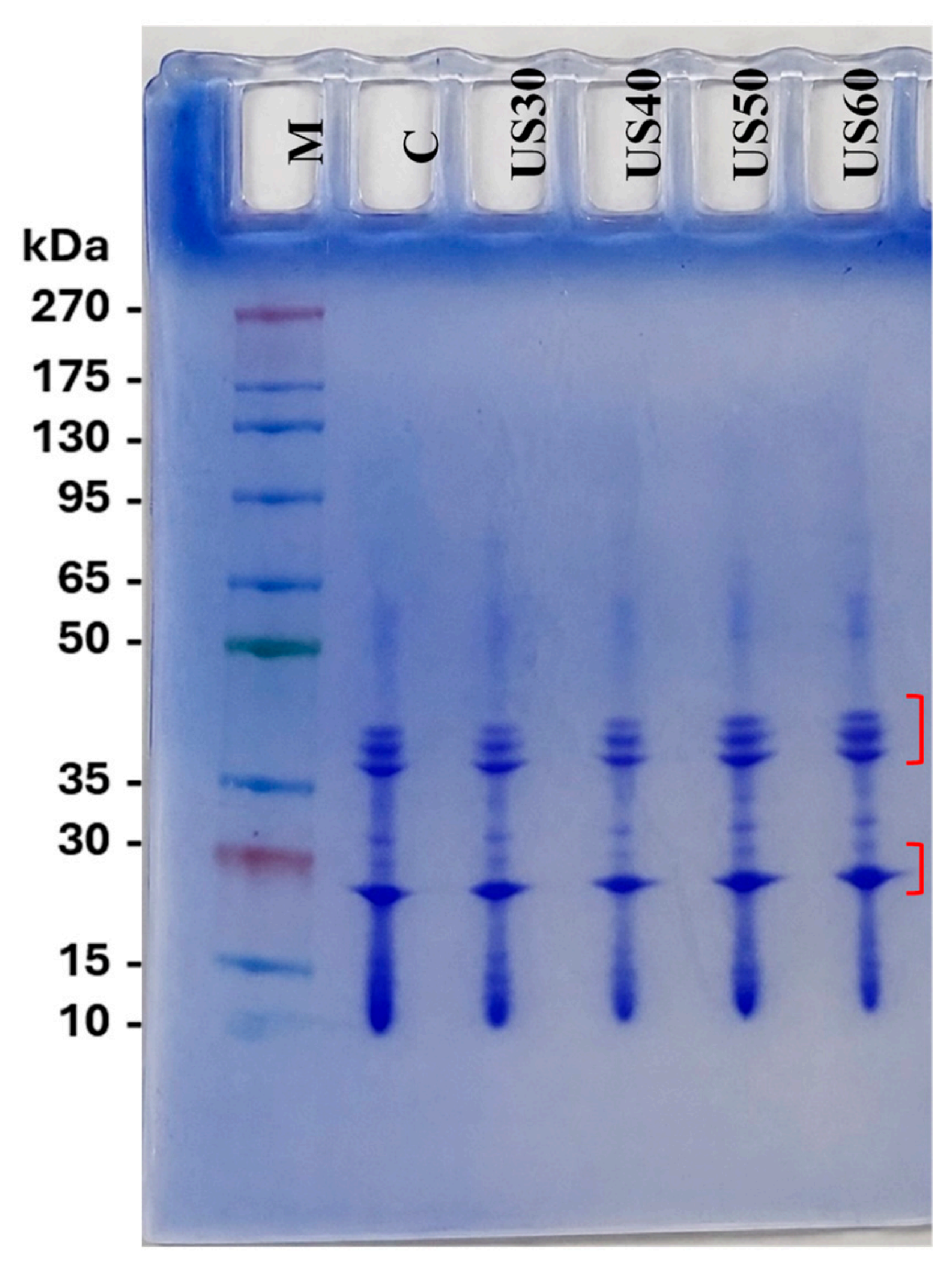
SDS–PAGE profiles of chicken feather keratin subjected to probe ultrasound treatment at different amplitudes. Lane M: molecular weight marker (10–270 kDa); lane C: control; lanes US30, US40, US50, and US60: keratin treated at 30, 40, 50, and 60% amplitude, respectively. The red brackets indicate the protein band regions at approximately 20–25 and 35–45 kDa.

The SDS–PAGE profiles of untreated and ultrasound-treated chicken feather keratin under reducing conditions are presented in Figure 3. All samples exhibited similar electrophoretic patterns, characterized by a prominent band at approximately 20–25 kDa, several less intense bands at approximately 35–45 kDa, and diffuse staining in the low-molecular-weight region. Comparable molecular-weight distributions have previously been reported for feather-derived keratin, including bands between 15 and 25 kDa [12] and keratin fractions of approximately 10 kDa [7]. The higher-molecular-weight bands may represent incompletely dissociated species or other co-extracted protein fractions; however, their identities cannot be established from SDS–PAGE alone.

Ultrasound treatment at amplitudes ranging from 30 to 60% did not produce marked shifts in the positions of the resolved bands or the appearance or disappearance of distinct bands upon visual inspection. Thus, the apparent molecular-weight profiles of the detectable protein fractions remained broadly similar under the electrophoretic conditions used. This observation is consistent with reports of ultrasound-induced changes in protein particle or aggregate size without substantial alterations in SDS–PAGE profiles [24]. Qin et al. [11] also reported that ultrasound enhanced feather disintegration and keratin solubility without causing pronounced structural degradation under optimized conditions. However, this qualitative similarity in band profiles does not exclude limited polypeptide-chain cleavage or changes in protein fractions below the resolution or detection limits of the analysis.

### 2.5. ζ-Potential and Surface Hydrophobicity (S_0_)

ζ-Potential reflects the net surface charge of a protein and is a key parameter affecting its solubility and colloidal stability in solution, whereas surface hydrophobicity (S_0_) indicates the exposure of nonpolar residues at the protein surface and contributes to interfacial behaviors, such as foaming and emulsifying activity [8]. The ζ-potential of control (untreated) and ultrasound-treated CFK samples was positive at pH 3.0 (34.77 ± 3.84 mV for the control) and negative at pH 5.0, 7.0, and 9.0 for all samples (Table 3). This pH-dependent charge reversal is consistent with the isoelectric point (pH 4.4) reported for keratin [6], below which keratin carries a net positive charge and above which it carries a net negative charge. On the other hand, Chuttke et al. [7], who extracted chicken feather keratin using a urea/L-cysteine-based method, reported a lower isoelectric point (pH 3.2) and a more strongly negative surface charge (approximately −40.4 mV) at neutral pH for keratin dispersion [7]. The different values reported suggest that the extraction method, rather than the feather source itself, influences the isoelectric behavior and surface charge of the resulting keratin.

Ultrasound treatment did not result in significant changes in ζ-potential at pH 5.0, 7.0, or 9.0. On the other hand, at pH 3.0, only the US60 sample differed significantly from the control, showing a lower positive charge (25.33 ± 1.59 mV). This finding indicates that ultrasound treatment at 30–60% amplitude left the surface charge behavior of keratin largely intact across the investigated pH range of 5.0-9.0, which is most relevant to food applications, consistent with the FTIR and SDS–PAGE results.

In contrast to ζ-potential, surface hydrophobicity (S_0_) showed no clear amplitude-dependent trend following ultrasound treatment. A statistically significant increase was observed only for US40 compared with the control (3.78 × 10^4^ vs. 2.19 × 10^4^, approximately 1.7-fold), whereas US30, US50, and US60 did not differ significantly from the control. A possible explanation is that cavitation at 40% amplitude exposed some buried hydrophobic residues, whereas stronger treatment led to reaggregation or rearrangement that hid them again. This explanation was not tested directly, since unfolding and aggregation were not measured. A non-linear relationship between ultrasound intensity or duration and protein structure was also reported by Qin et al. [11], and Raydan et al. [13] described threshold-like folding and unfolding of feather keratin under different processing conditions.

### 2.6. Antioxidant Activity

**Figure 4:**
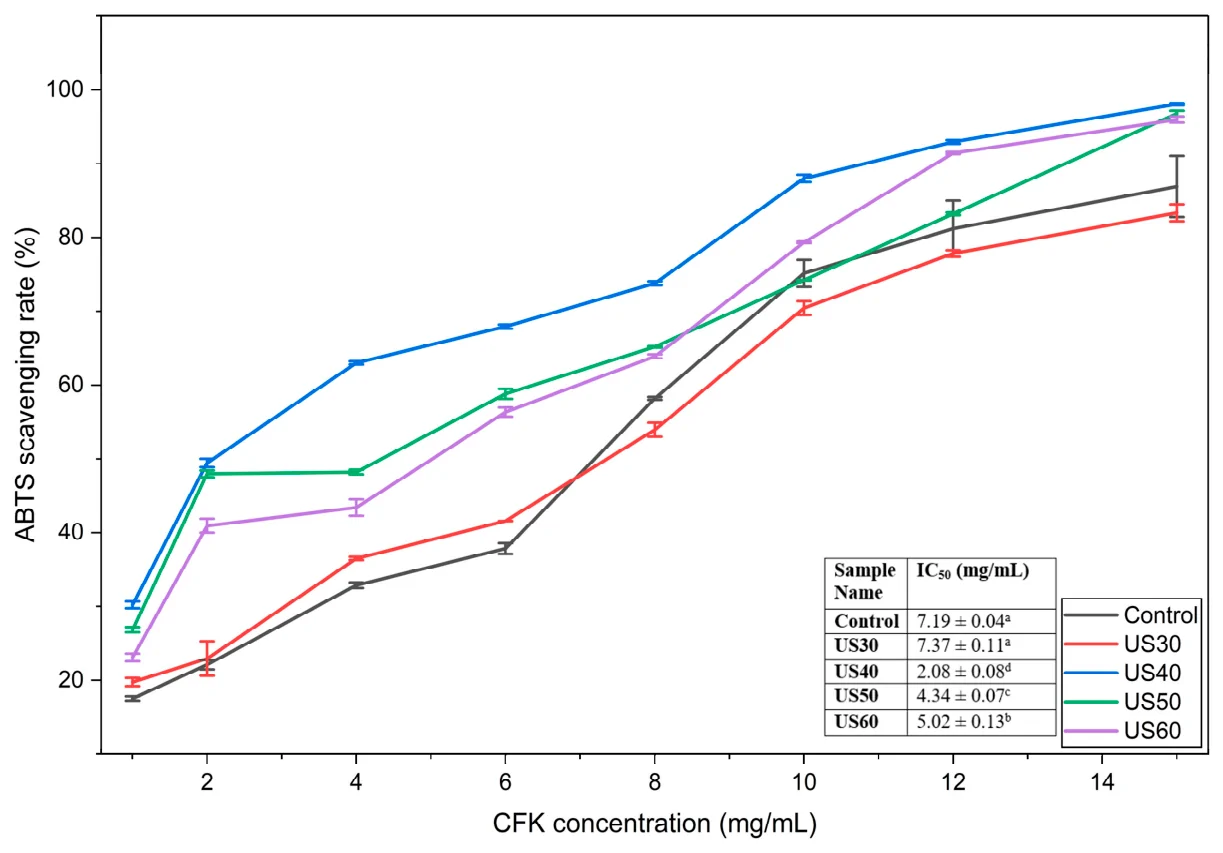
ABTS radical scavenging activity of control (untreated) and ultrasound-treated chicken feather keratin (CFK) as a function of CFK concentration. Data points represent mean values ± standard deviation. The inset table presents IC_50_ values expressed as mean ± standard deviation. Different lowercase superscript letters indicate significant differences among samples (*p* < 0.05). Control represents untreated CFK, while US30, US40, US50, and US60 represent CFK samples treated with ultrasound at amplitudes of 30, 40, 50, and 60%, respectively.

Antioxidant activity is a valuable functional property of proteins and protein fractions, relevant not only to food preservation and the inhibition of lipid oxidation but also to the growing use of keratin-derived ingredients as natural antioxidants in health-oriented applications [8]. The antioxidant activity of the control (untreated) and ultrasound-treated CFK samples was evaluated using two complementary assays, ABTS and CUPRAC, which differ in their underlying reaction mechanisms. The ABTS radical scavenging activity of untreated and ultrasound-treated CFK samples increased with increasing CFK concentration for all samples (Figure 4). The highest scavenging activity (~98%) at 15 mg/mL was reached by the US40 sample, followed by US50 (~97%) and US60 (~96%), while the control (~87%) and US30 (~83%) samples reached the lowest scavenging levels.

The IC_50_ values (the concentration required for 50% radical inhibition) showed the same trend. The lowest (in other words most potent) IC_50_ value was obtained for US40 (2.08 ± 0.08 mg/mL), followed by US50 (4.34 ± 0.07 mg/mL) and US60 (5.02 ± 0.13 mg/mL), all significantly lower than the control (7.19 ± 0.04 mg/mL). US30 (7.37 ± 0.11 mg/mL) did not differ significantly from the control, whereas significant differences were observed among US40, US50, and US60. Overall, it can be concluded that ultrasound treatment at 40–60% amplitude significantly enhanced the ABTS-based antioxidant capacity of feather keratin, with the greatest improvement observed at 40% amplitude.

A comparable, amplitude-dependent relationship between ultrasound treatment and antioxidant activity has been reported for other protein systems. For instance, in soy protein isolate nanofibrils, low-amplitude ultrasound improved ABTS and DPPH radical scavenging activity, whereas high-amplitude treatment reduced this activity. This finding was attributed to protein unfolding exposing previously buried aromatic and sulfur-containing residues at moderate intensity, and to structural damage or reaggregation limiting this exposure at higher intensity [14]. In the present study, ABTS activity was highest at 40% amplitude, and was relatively lower at 50–60%, whereas 30% had no significant effect. The amplitudes are not directly comparable between studies, because the proteins, probes, power, and treatment times differ. Therefore, the present results are consistent with previous observations that the effects of ultrasound treatment on protein antioxidant activity may not increase proportionally with treatment intensity, as also reported for keratin structure in relation to ultrasound intensity and duration [11].

The IC_50_ values obtained (2.08–7.37 mg/mL) indicate relatively lower potency compared to the value reported for enzymatically hydrolyzed pig hoof keratin in the ABTS assay (0.96 ± 0.01 mg/mL) [8], yet they fall within the broader IC_50_ range (0.53–8.21 mg/mL) previously reported for keratin extracted by different chemical methods [25,26,27]. The observed difference can be attributed to the fact that the keratin samples in the present study were only physically modified by ultrasound treatment rather than enzymatically hydrolyzed. Enzymatic hydrolysis has been reported to significantly decrease peptide size, which is generally associated with enhanced radical-scavenging activity [8]. Direct comparison is limited by differences in assay conditions and in the basis used to express concentration.

**Figure 5:**
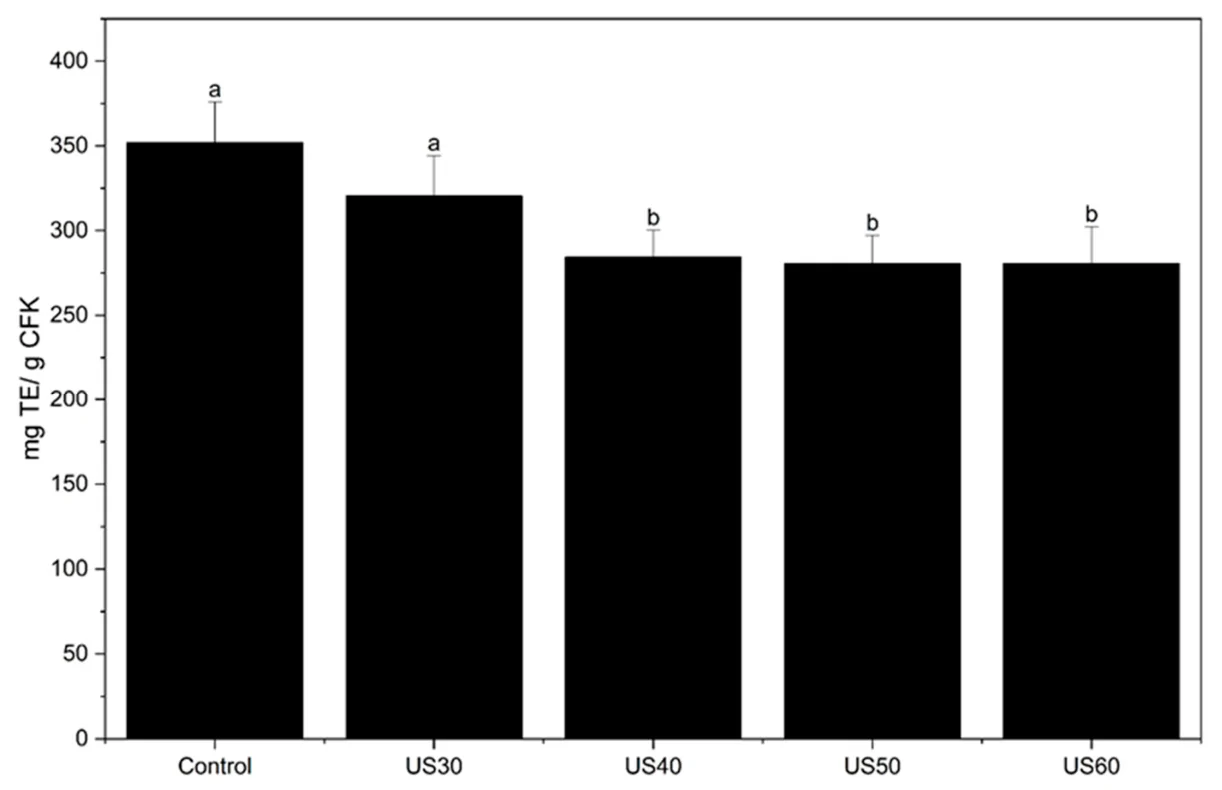
Cupric reducing antioxidant capacity (CUPRAC) of control (untreated) and ultrasound-treated chicken feather keratin (CFK) samples, expressed as mg Trolox equivalents (TE) per g CFK. Values are presented as mean ± standard deviation. Different lowercase superscript letters indicate significant differences among samples (*p* < 0.05). Control represents untreated CFK, while US30, US40, US50, and US60 represent CFK samples treated with ultrasound at amplitudes of 30, 40, 50, and 60%, respectively.

Unlike the ABTS results, the cupric reducing antioxidant capacity (CUPRAC) values showed the opposite trend with respect to ultrasound treatment (Figure 5). The control sample showed the highest copper-reducing capacity (~352 mg TE/g CFK), and the US30 sample did not differ significantly from the control (~320 mg TE/g CFK). On the other hand, US40, US50, and US60 samples showed significantly lower CUPRAC values than the control (~280–284 mg TE/g CFK), with no significant differences among these three treated samples. The different responses observed in the ABTS and CUPRAC assays (an improvement in radical-scavenging activity but a reduction in copper-reducing capacity at 40–60% ultrasound amplitude) can be attributed to the fact that the two assays measure different aspects of antioxidant activity. The ABTS assay involves a mixed hydrogen-atom-transfer/single-electron-transfer reaction with a pre-formed radical cation, whereas CUPRAC is a purely electron-transfer-based assay that measures the ability of a sample to reduce Cu(II) to the Cu(I)-neocuproine chromophore under different reaction conditions including pH, kinetics, and reagent chemistry [28]. The structural changes induced by ultrasound (Section 2.2 and Section 2.3) may affect the two assays differently, improving the response in one while having a minor or even a negative effect in the other. These differences between assays highlight the importance of using more than one method for evaluating antioxidant activity. Therefore, both ABTS and CUPRAC were used in the present study to provide a more comprehensive assessment of ultrasound-treated keratin.

The change in free –SH content (Section 2.3) did not parallel the antioxidant results. Free –SH had decreased by ~70% at 30% amplitude, whereas the ABTS IC_50_ improved only at 40–60% amplitude and CUPRAC decreased only at 40–60% amplitude. Moreover, US60, which had the lowest free –SH content, did not show the lowest IC_50_. Free –SH groups are known to contribute to the antioxidant activity of keratin [26], but thiol loss alone did not predict the activity of the sonicated samples. Other changes caused by ultrasound, such as protein unfolding and the exposure of previously buried residues [14], may also contribute.

### 2.7. In Vitro Protein Digestibility

**Figure 6:**
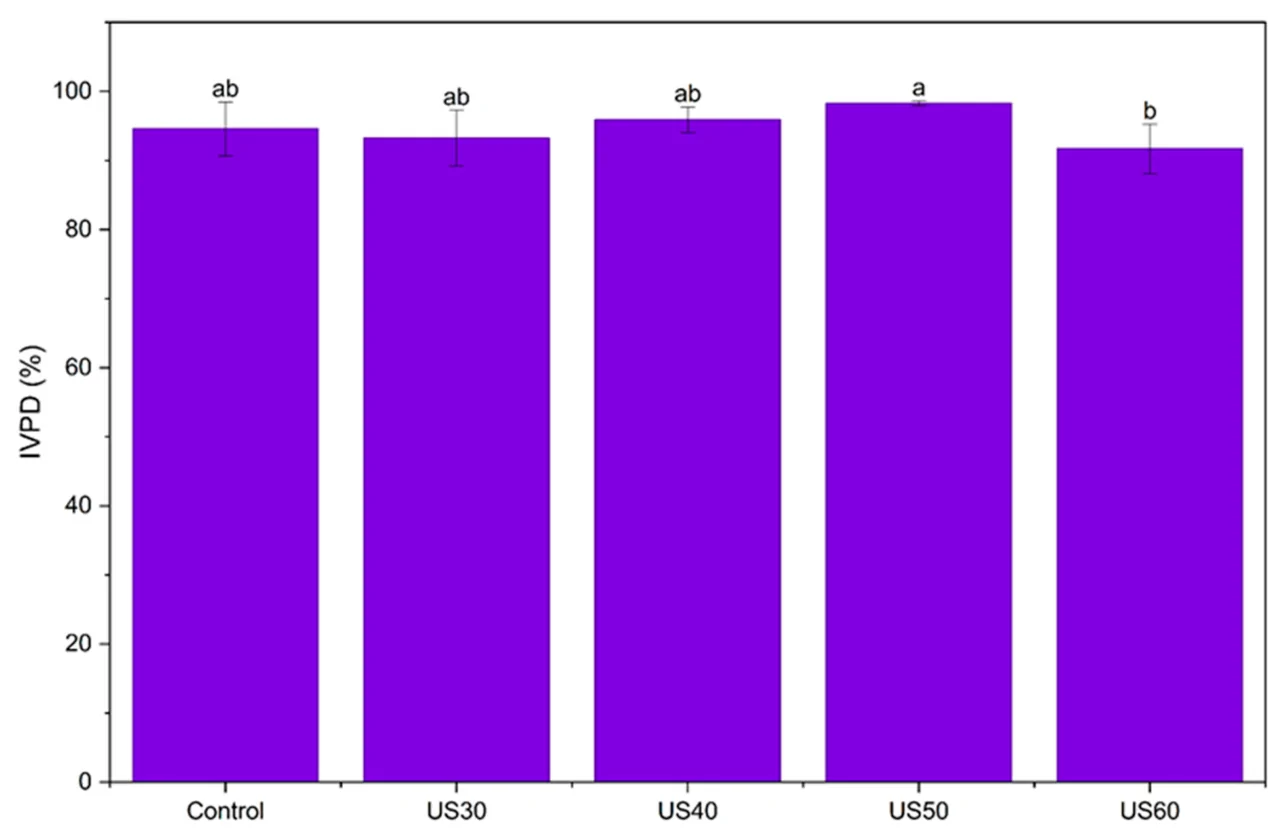
In vitro protein digestibility (IVPD) of control (untreated) and ultrasound-treated chicken feather keratin (CFK) samples. Values are presented as mean ± standard deviation. Different lowercase superscript letters indicate significant differences among samples (*p* < 0.05). Control represents untreated CFK, while US30, US40, US50, and US60 represent CFK samples treated with ultrasound at amplitudes of 30, 40, 50, and 60%, respectively.

Native keratin is inherently resistant to chemical, physical, and enzymatic degradation due to its dense disulfide cross-links [4,26]. However, in the present study, all samples including the untreated control showed in vitro protein digestibility (IVPD) values above 90% (Figure 6). This finding suggests that the alkaline hydrolysis used for keratin extraction disrupted the disulfide and hydrogen-bond interactions responsible for keratin’s natural recalcitrance to degradation [3,4], resulting in a more soluble and digestible protein fraction, consistent with values reported for other enzymatically or chemically processed keratins [8].

Among the treated samples, US50 showed the highest IVPD (98.3%), which was numerically higher than the control (94.6%) and significantly higher than US60 (91.7%). The control, US30, and US40 samples did not differ significantly from one another or from either US50 or US60. The lower IVPD of US60 was significant only in comparison with US50 and did not differ from the control. The reason for this difference was not examined, since aggregate size and enzyme accessibility under digestion conditions were not measured. The IVPD values refer to the soluble protein fraction determined by the Bradford assay, which responds weakly to small peptides. Therefore, they should be interpreted as relative values for comparing the samples rather than as absolute digestibility. Since IVPD remained >90% in all samples, it can be concluded that ultrasound treatment at 30–60% amplitude did not adversely affect the digestibility of the extracted keratin.

### 2.8. Water Solubility

**Figure 7:**
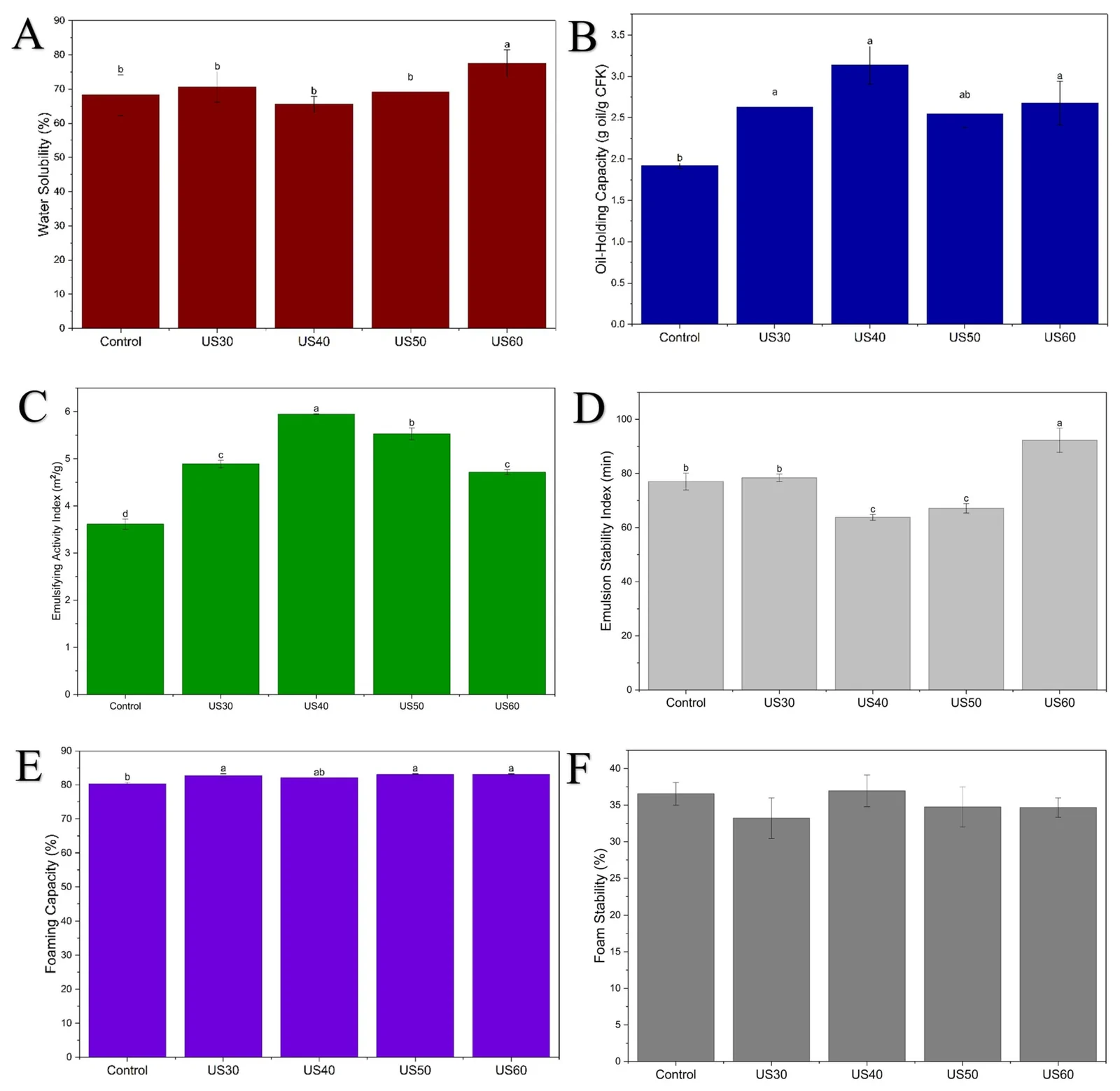
Techno-functional properties of control (untreated) and ultrasound-treated chicken feather keratin (CFK) samples: (**A**) water solubility, (**B**) oil-holding capacity (OHC), (**C**) emulsifying activity index (EAI), (**D**) emulsion stability index (ESI), (**E**) foaming capacity (FC), and (**F**) foam stability (FS). Values are presented as mean ± standard deviation. Different lowercase superscript letters indicate significant differences among samples (*p* < 0.05). Control represents untreated CFK, while US30, US40, US50, and US60 represent CFK samples treated with ultrasound at amplitudes of 30, 40, 50, and 60%, respectively.

The water solubility of the control (untreated) and ultrasound-treated CFK samples ranged from approximately 65% to 78% (Figure 7A). US60 (~78%) showed significantly higher solubility than all other samples, including the control (~68%). On the other hand, US30 (~71%), US40 (~66%), and US50 (~69%) were found to be similar (*p* > 0.05). The higher solubility of US60 cannot be explained directly by the ζ-potential or surface hydrophobicity results (Section 2.5). The ζ-potential at pH 7.0, the pH at which solubility was measured, did not differ among the samples, and the surface hydrophobicity of US60 was not different from that of the control. The ζ-potential of US60 differed from the control only at pH 3.0. Ultrasound has been reported to reduce the size of protein particles or aggregates [24], which could increase the soluble fraction, but particle size was not measured in this study. The reason for the higher solubility of US60 therefore remains to be determined.

Water solubility is a prerequisite for many of the other functional properties examined in this study, since a protein must first disperse and hydrate before it can adsorb at an air–water or oil–water interface, stabilize a foam lamella, or contribute to gel or film formation [29]. Hydrolyzed keratin fractions with sufficient solubility have been reported to show surface-active, emulsifying, and thickening properties that are mainly absent in poorly soluble, high-molecular-weight keratin [9]. The improved solubility observed for US60 may influence its emulsifying and foaming behavior, as examined in Section 2.10.

Water-soluble keratin fractions are already established ingredients in the cosmetics and personal care industry, where they are recognized under the INCI name “Hydrolyzed Keratin” and used as skin-moisturizing, hair-repairing, nail-hardening, and conditioning agents in a wide range of formulations [9]. Since solubility is an important consideration for incorporation into such formulations, the relatively high solubility observed at 60% ultrasound amplitude may be of interest for food and other applications. However, the suitability of the ultrasound-treated keratin for any specific formulation was not tested in this study.

### 2.9. Oil-Holding Capacity

Oil-holding capacity reflects a protein’s ability to physically entrap and retain lipids within its matrix, an important property in food systems for improving the texture, juiciness, and flavor retention of meat and bakery products [8], and is mechanistically related to the emulsifying and thickening behaviors in cosmetic and personal-care formulations [9].

Oil-holding capacity (OHC) increased significantly in all ultrasound-treated samples relative to the control (1.9 g oil/g CFK) (Figure 7B). The highest value was obtained for US40 (3.1 g/g), followed by US60 (2.7 g/g) and US30 (2.6 g/g), whereas US50 (approximately 2.55 g/g) fell into an intermediate statistical group, not significantly different from either the control or the higher-performing treated samples.

US40 had both the highest S_0_ (Table 3) and the highest OHC. However, S_0_ increased significantly only at US40, whereas OHC increased in all ultrasound-treated samples, so S_0_ alone cannot explain the OHC results. Other factors, such as changes in aggregate size or particle structure, may also play a role, but these parameters were not measured in the present study. On the other hand, Wang et al. [8] reported the opposite trend for hydrolyzed pig hoof keratin, which showed relatively low surface hydrophobicity but high OHC. The authors suggested that this difference may be related to peptide size and density rather than ANS-detectable surface hydrophobicity. In conclusion, surface hydrophobicity along with other structural changes may contribute to the oil-binding behavior of ultrasound-treated feather keratin.

Furthermore, the OHC values obtained in our study (1.9–3.1 g/g) remain significantly lower than the values reported for enzymatically hydrolyzed pig hoof keratin (4.35 ± 0.20 g/g) [8]. This difference may be related to the reduction in peptide size caused by enzymatic hydrolysis, increasing the accessibility of hydrophobic sites for oil binding, an effect not achieved by the physical (ultrasound-only) modification applied in the present study. Particularly, the control value obtained (1.9 g/g) was very close to that reported for native, unhydrolyzed pig hoof keratin (1.91 ± 0.18 g/g) [8], indicating similar oil-holding capacity between the two keratin sources prior to any modification.

### 2.10. Emulsifying and Foaming Properties

Emulsifying and foaming properties are key techno-functional properties that determine a protein’s ability to adsorb at an interface and stabilize a dispersed phase, whether oil droplets in an emulsion or air bubbles in a foam. These properties are important for applications of proteins in a wide range of food systems, including dressings, sauces, whipped toppings, bakery and beverage formulations. Emulsifying and foaming properties are also relevant beyond food applications in the industry. For instance, water-soluble keratin hydrolysates are already recognized as natural emulsifying and foam-forming agents in pharmaceutical and cosmetic formulations such as creams, lotions, and cleansing foams [9].

The emulsifying activity index (EAI) increased significantly in all ultrasound-treated samples relative to the control (3.61 m^2^/g) (Figure 7C). The highest value was obtained at US40 (5.95 m^2^/g), followed by US50 (5.53 m^2^/g), while US30 and US60 formed a lower, statistically similar group (4.89 and 4.71 m^2^/g, respectively). In contrast, the emulsion stability index (ESI) showed a significantly different trend (Figure 7D). US60 achieved the highest stability (92.3 min), while US40 and US50 (the two samples with the highest EAI) showed significantly lower stability than the control (63.8 and 67.1 min, respectively). This finding suggests that US40, although showing the highest initial emulsifying performance, produced one of the least stable emulsions over time. In contrast, US60 showed better emulsion stability despite its lower initial performance.

Perinelli et al. [9] reported that the emulsifying capacity of commercial hydrolyzed keratins is related to molecular weight, with fractions above 3000 Da showing significantly higher emulsifying performance. Since the SDS–PAGE band profiles were similar among the samples (Figure 3), the differences in EAI and ESI may be related to conformational rearrangements (Table 2), surface hydrophobicity, and solubility rather than to major changes in molecular weight, although limited chain cleavage cannot be excluded. US40 had the highest S_0_ (Table 3), which may be related to its high EAI, and its low ESI may reflect droplet flocculation or coalescence over time. The highest ESI observed at US60 is in line with its highest water solubility (Figure 7A). However, EAI and ESI did not follow S_0_ or the β-sheet content consistently across the amplitudes. For example, US50 had among the lowest β-sheet contents (Table 2) but low emulsion stability. Since droplet size and interfacial behavior were not directly measured, the role of ultrasound-induced conformational changes in these functional differences could not be confirmed and requires further study.

Foaming capacity was significantly higher in US30, US50, and US60 than in the control (80%), reaching 82.7–83% (Figure 7E), while foam stability showed no significant differences among the samples, remaining within the 33–37% range (Figure 7F). This finding indicates that ultrasound treatment slightly improved the ability of keratin to incorporate air during whipping, without changing the stability of the resulting foam over time. Babaniyi et al. [30] reported that foam stability in extracted keratins can change significantly with the extraction method and solvent system used. In the present study, all samples were prepared using the same alkaline-hydrolysis protocol and differed only in the subsequent ultrasound treatment. The absence of a significant change in foam stability across the tested amplitudes may therefore be related to the consistent extraction conditions used for all samples.

Overall, these results suggest that ultrasound treatment, particularly at 50–60% amplitude, can enhance the emulsion-forming and foam-forming capacity of feather keratin without adversely affecting foam stability. On the other hand, relatively lower emulsion stability observed at US40 suggests that these functional properties do not necessarily improve in parallel. Soluble keratin hydrolysates are already used as emulsifying and foaming agents in cosmetic and personal-care formulations [9]. Whether ultrasound-treated feather keratin can perform similarly in such products was not tested here, because no formulation, stability, safety, or performance tests were carried out.

## 3. Materials and Methods

### 3.1. Materials

Chicken feathers were supplied by Senpilic Gida San. A.S. (Geyve, Sakarya, Türkiye), while sunflower oil was purchased from a local market. Pepsin (541 U/mg; EC 3.4.23.1), pancreatin (8× USP specifications), bovine serum albumin (BSA), potassium phosphate monobasic, 2,2′-azino-bis(3-ethylbenzothiazoline-6-sulfonic acid) (ABTS), potassium persulfate, neocuproine, 8-anilino-1-naphthalenesulfonic acid (ANS), Trolox, and sodium dodecyl sulfate (SDS) were purchased from Sigma-Aldrich (Darmstadt, Germany). Sodium hydroxide (NaOH), hydrochloric acid (HCl), sodium carbonate (Na_2_CO_3_), copper(II) chloride, ammonium acetate, absolute ethanol (99%), methanol, and glacial acetic acid were purchased from ISOLAB (Eschau, Germany). Bradford reagent was purchased from SERVA Electrophoresis GmbH (Heidelberg, Germany).

### 3.2. Extraction of Keratin from Chicken Feathers

Keratin was extracted from feathers by alkaline hydrolysis according to the method described by Mengistu et al. [5], with slight modifications. The extraction conditions, including NaOH concentration, temperature, and treatment time, were selected based on preliminary trials. Briefly, approximately 20 g of feather samples were immersed in 250 mL of 1.0 N NaOH solution and incubated at 50 °C for 1 h. Isoelectric precipitation was then performed by adjusting the pH of the hydrolysate to 4.4 with 2.0 M HCl. The precipitated keratin was separated by centrifugation at 8600× *g* for 5 min, freeze-dried without washing, packaged in airtight containers, and stored at 4 °C until further analysis.

### 3.3. Determination of Extraction Yield and Protein Recovery

The extraction yield of chicken feather keratin (CFK) was determined as the ratio of the dry mass of the recovered, freeze-dried keratin extract to the initial dry mass of chicken feathers, expressed as a percentage. The crude protein contents of the initial chicken feathers and recovered CFK extract were determined using the Kjeldahl method. The total nitrogen content was converted to crude protein content using a nitrogen-to-protein conversion factor of 6.25, as previously reported for feather-derived keratin by Mengistu et al. [5]. Protein recovery was calculated as the percentage of the initial crude protein mass in the chicken feathers recovered in the freeze-dried CFK extract. Extraction yield and protein recovery were calculated using the following equations:

(1) $$Crude\;protein\;\left(\%\right)=\;Total\;nitrogen(\%)\times 6.25$$

(2) $$Extraction\;yield\;(\%)=\frac{m_{e}}{m_{f}}\times 100$$

(3) $$Protein\;recovery\;(\%)=\frac{m_{e}\times P_{e}}{m_{f}\times P_{f}}\times 100$$

where *m_e_* is the dry mass of the recovered, freeze-dried CFK extract (g), *m_f_* is the initial dry mass of chicken feathers (g), *P_e_* is the crude protein mass fraction of the recovered CFK extract, and *P_f_* is the crude protein mass fraction of the initial chicken feathers.

### 3.4. Ultrasonic Treatment of Chicken Feather Keratin

For the ultrasound modification of CFK, CFK solutions were prepared at a concentration of 50 mg/mL in 10 mM phosphate-buffered saline (PBS) (pH 7.0). A total volume of 250 mL of each dispersion was treated using a SONOPULS HD 2200 probe-type ultrasonic homogenizer (BANDELIN electronic GmbH & Co. KG, Berlin, Germany), operating at a frequency of 20 kHz with a maximum nominal ultrasonic power of 200 W and equipped with a 13 mm diameter titanium probe. Ultrasonication was performed at amplitude settings of 30, 40, 50, and 60% for a total treatment time of 10 min, using a pulse mode of 2 s on and 2 s off, corresponding to an effective sonication time of 5 min. The power actually delivered to the sample was not measured; the nominal power given above is the maximum rating of the instrument, not the energy input to the dispersion. During treatment, the sample beaker was placed in an ice bath to minimize temperature increases. The alkali-extracted CFK that was not treated with ultrasound was referred to as the “control (untreated) CFK”, in short, “control”. Following ultrasonication, the CFK dispersions were transferred into Petri dishes and freeze-dried without an additional washing step. To ensure comparable sample preparation conditions, the control sample was also dispersed in 10 mM PBS (pH 7.0) at the same CFK concentration and freeze-dried under identical conditions without ultrasound treatment.

### 3.5. Characterization of Chicken Feather Keratin

#### 3.5.1. Fourier Transform Infrared Spectroscopy and Secondary Structure Analysis

Structural differences between control (untreated) CFK and ultrasound-treated CFK samples were investigated using Fourier transform infrared (FTIR) spectroscopy (IRTracer-100, Shimadzu, Kyoto, Japan). Samples in powder form were used for the analysis, and the spectra were recorded in transmittance mode from 750 to 4000 cm^−1^ at a resolution of 4 cm^−1^ using 15 scans. The secondary structure of keratin was quantitatively evaluated by curve-fitting the Amide I region (1600–1700 cm^−1^) using OriginPro 2026b (OriginLab Corp., Northampton, MA, USA). After baseline correction and Savitzky–Golay smoothing, second-derivative analysis was used to determine peak centers. Gaussian deconvolution was performed using the Levenberg–Marquardt algorithm, and the relative percentage of each conformation was calculated from the integrated area of each peak relative to the total Amide I.

#### 3.5.2. Determination of Free Sulfhydryl (–SH) Group

The free sulfhydryl (–SH) group content of untreated and ultrasound-treated chicken feather keratin (CFK) samples was determined using 5,5′-dithiobis(2-nitrobenzoic acid) (DTNB, Ellman’s reagent), according to the method described by Xie et al. [31], with slight modifications. Briefly, CFK samples were prepared at a concentration of 3 mg/mL in Tris–glycine buffer (0.086 M Tris, 0.09 M glycine, and 4 mM EDTA, pH 8.0). Approximately 0.5 mL of each sample solution was mixed with 50 µL of DTNB solution (4 mg/mL, prepared in the same buffer), followed by the addition of 2 mL of Tris–glycine buffer. The mixtures were vortexed for 10 s and incubated at room temperature for 15 min. Sample blanks were prepared without DTNB to account for background absorbance from the CFK samples. The absorbance was then measured at 412 nm using a Multiskan SkyHigh Microplate Spectrophotometer (Thermo Fisher Scientific, Vantaa, Finland). The free sulfhydryl (–SH) group content was calculated using the following equation and expressed as µmol/g of protein:

(4) $$\mathrm{SH}\;(\mu \mathrm{mol}/g)\;=\;\frac{{73.53\times A}_{412}\times D}{C\times l}$$

where *A*_412_ is the blank-corrected absorbance at 412 nm, 73.53 is the conversion factor derived from the molar extinction coefficient of TNB (13,600 M^−1^ cm^−1^), *D* (5.1) is the dilution factor calculated from the total reaction volume (2.55 mL) and sample volume (0.5 mL), *C* is the initial protein concentration (mg/mL), and *l* is the optical path length (cm).

#### 3.5.3. SDS-PAGE Analysis

The molecular weight distribution of the protein samples was evaluated by sodium dodecyl sulfate–polyacrylamide gel electrophoresis (SDS-PAGE) according to Karabulut et al. [32], with minor modifications. Protein concentrations were determined using the Bradford assay with BSA as the calibration standard, and samples were adjusted to an equal final protein concentration of 5 mg/mL in 1× sample buffer containing β-mercaptoethanol. After denaturation at 90 °C for 10 min, 10 µL of each sample, corresponding to 50 µg of protein per lane, was loaded onto 4–20% ExpressPlus™ precast polyacrylamide gels (GenScript, Piscataway, NJ, USA). Broad Multi Color Pre-Stained Protein Standard (10–270 kDa; GenScript, USA) was used as the molecular weight marker. Electrophoresis was performed at 90 V until the tracking dye reached approximately the midpoint of the gel, after which the voltage was increased to 120 V and maintained until the dye front approached the bottom of the gel. The gels were stained overnight with 0.05% (*w*/*v*) Coomassie Brilliant Blue R-250 in 25% (*v*/*v*) methanol and 5% (*v*/*v*) acetic acid. Destaining was carried out for 45 min using 30% (*v*/*v*) methanol and 10% (*v*/*v*) acetic acid, with the solution replaced at 15-min intervals.

#### 3.5.4. ζ-Potential

The ζ-potential of the CFK solutions was determined using a Zetasizer Nano ZS90 instrument (Malvern Instruments Ltd., Worcestershire, UK). A 1% (*w*/*v*) CFK solution was prepared in 10 mM PBS (pH 7.0), diluted 10-fold, and adjusted to pH 3.0, 5.0, 7.0, and 9.0 using 0.5 M HCl or 0.5 M NaOH. Measurements were performed at 20 °C.

#### 3.5.5. Surface Hydrophobicity

The surface hydrophobicity (S_0_) of the keratin samples was determined according to the method described by Wang et al. [8]. Dispersions of control (untreated) and ultrasound-treated CFK were prepared at concentrations ranging from 0.002 to 0.010% in 10 mM phosphate buffer (pH 7.00), with a final volume of 4 mL. Afterwards, 20 µL of an 8 mM 8-anilino-1-naphthalenesulfonic acid (ANS) solution prepared in the 10 mM phosphate buffer (pH 7.00) was added to each sample. The mixtures were incubated in the dark at room temperature for 15 min. Fluorescence intensity was then measured using a spectrofluorometer (SPEX Fluorolog 3-2iHR, Horiba Scientific, Edison, NJ, USA) at excitation and emission wavelengths of 390 and 470 nm, respectively. Phosphate buffer containing ANS and corresponding protein solutions without ANS were used as blanks for background correction. The fluorescence intensity values were plotted against protein concentration, and the slope of the resulting linear regression curve was taken as the surface hydrophobicity value (S_0_).

#### 3.5.6. Antioxidant Capacity

The antioxidant activities of control (untreated) and ultrasound-treated CFK were determined using the 2,2′-azino-bis(3-ethylbenzothiazoline-6-sulfonic acid) (ABTS) and cupric reducing antioxidant capacity (CUPRAC) assays.

The ABTS assay was performed according to the method described by Zeng et al. [33], with slight modifications. The ABTS stock solution was prepared by mixing 7 mM ABTS solution with 2.45 mM potassium persulfate solution and allowing the mixture to react in the dark for 16 h. The resulting ABTS radical cation solution was diluted with 10 mM phosphate-buffered saline (PBS; pH 7.0) until an absorbance of 0.700 ± 0.020 at 734 nm was obtained using a Multiskan SkyHigh microplate reader (Thermo Fisher Scientific, Vantaa, Finland). Dispersions of control (untreated) and ultrasound-treated CFK were prepared in PBS at concentrations ranging from 1 to 15 mg/mL. Subsequently, 100 µL of each sample dispersion was mixed with 200 µL of the ABTS working solution and incubated in the dark at 30 °C for 6 min. For each sample concentration, a corresponding sample blank was prepared by replacing the ABTS working solution with PBS. The control was prepared by mixing 100 µL of PBS with 200 µL of the ABTS working solution. The ABTS radical-scavenging activity of CFK was calculated as a percentage using the following equation:

(5) $$ABTS\;Radical\;Scavenging\;Activity\;(\%)=\left[1-\frac{A_{sample}-A_{blank}}{A_{control}}\right]\times 100$$

where A*_control_*, A*_sample_* and A*_blank_* represent the absorbance values of the ABTS working solution mixed with PBS, the CFK solution mixed with the ABTS working solution, and the CFK solution mixed with PBS (without ABTS), respectively.

The IC_50_ value, defined as the sample concentration required to achieve 50% ABTS radical scavenging activity, was determined by linear interpolation between the two experimental concentrations immediately below and above 50% inhibition.

The CUPRAC assay was performed by using the method of Apak et al. [34]. In brief, 0.25 mg/mL CFK solution was prepared in PBS (10 mM, pH 7.00) and 100 µL of CFK solution was mixed with 1 mL each of 1.0 M ammonium acetate buffer (pH 7.00), 7.5 mM neocuproine in ethanol, 10 mM copper (II) chloride solution, and distilled water. After 30 min of incubation in the dark, measurements were performed at 450 nm by using a Multiskan SkyHigh microplate reader (Thermo Fisher Scientific, Waltham, MA, USA). Results were presented as mg Trolox equivalent (TE)/g CFK.

#### 3.5.7. In Vitro Protein Digestibility Measurement

The in vitro protein digestibility (IVPD) of control (untreated) and US-treated CFK samples was determined according to the method described by Rout and Srivastav [35], with slight modifications. Briefly, 0.5 g of each CFK sample was dispersed in 20 mL of 10 mM PBS (pH 7.0). To simulate gastric conditions, the pH of the dispersion was adjusted to 1.5, followed by the addition of 50 µL of pepsin solution (10 mg/mL; 541 U/mg), supplying 0.5 mg of enzyme preparation and 270.5 U per reaction. The samples were incubated in a water bath at 37 °C for 30 min. At the end of the gastric digestion stage, the pH was adjusted to 7.8 using 1 M NaOH to initiate the intestinal digestion stage. Subsequently, 200 µL of pancreatin solution (10 mg/mL; 8× USP specifications) was added, supplying 2 mg of enzyme preparation per reaction. The samples were incubated at 40 °C for 60 min, after which 50 µL of 150 mM Na_2_CO_3_ solution was added. The pepsin- and pancreatin-preparation-to-CFK powder ratios were 1:1000 and 1:250 (*w*/*w*), respectively.

Soluble protein concentrations before and after digestion were determined using the Bradford assay, with BSA as the calibration standard. The post-digestion protein concentration was corrected by subtracting the enzyme-only blank. Concentrations were corrected for the dilutions applied before the Bradford assay. IVPD was calculated using the following equation:

(6) $$IVPD(\%)=\frac{Initial\;protein\;concentration-Undigested\;protein\;concentration}{Initial\;protein\;concentration}\times 100$$

#### 3.5.8. Water Solubility Measurement

The protein solubility of CFK was determined according to the methods described by Miranda et al. [36], with slight modifications. CFK dispersions were prepared at a concentration of 10 mg/mL in 10 mM PBS (pH 7.0). The dispersions were centrifuged at 8600× *g* for 10 min, and the soluble protein content in the resulting supernatant was determined using the Bradford assay, with BSA as the calibration standard. Protein solubility was expressed as the percentage ratio of the protein mass in the supernatant to the initial protein mass in the sample.

#### 3.5.9. Oil-Holding Capacity Measurement 

The oil-holding capacity (OHC) of CFK was determined according to the method described by Wang et al. [8], with slight modifications. Briefly, 0.5 g of CFK (m_0_) was weighed into a centrifuge tube, and the combined mass of the tube and CFK was recorded (m_1_). Then, 5 g of sunflower oil was added, and the mixture was vortexed for 20 min. The mixture was then centrifuged at 8600× *g* for 20 min. Following centrifugation, the unabsorbed oil was decanted, and the tube was weighed again (m_2_). The OHC was calculated using the following equation:

(7) $$OHC\;(g\;oil/g\;CFK)=\frac{m_{2}-m_{1}}{m_{0}}$$

where m_0_ is the mass of CFK (0.5 g), m_1_ is the combined mass of the centrifuge tube and CFK before oil addition, and m_2_ is the mass of the tube after decanting the unabsorbed oil.

#### 3.5.10. Emulsifying and Foaming Properties Measurements

The emulsifying and foaming properties of CFK were determined according to the method described by Wang et al. [8], with slight modifications. To determine the emulsifying activity index (EAI) and emulsifying stability index (ESI), CFK solutions were prepared at a concentration of 10 mg/mL in 10 mM PBS (pH 7.0). Approximately 2 mL of sunflower oil and 8 mL of CFK solution were homogenized using an Ultra-Turrax homogenizer (IKA Ultra-Turrax T18, Staufen, Germany) at 10,000 rpm for 1 min. Immediately after homogenization, 50 µL of the emulsion was diluted with 4.5 mL of 0.1% SDS solution, and the absorbance was measured at 500 nm (A_0_). The optical path length was accounted for in the EAI calculation by using the corresponding path length of the absorbance measurement. After 30 min, another 50 µL aliquot of the emulsion was diluted with 4.5 mL of 0.1% SDS solution, and the absorbance was measured again (A_30_). The EAI and ESI were calculated using the following equations:

(8) $$EAI\;(m^{2}/g)=\frac{2\times 2.303\times A_{0}}{\phi \times ∁\times 100}$$

(9) $$ESI\;(min)=\frac{A_{0}}{A_{0}-A_{30}}\times 30$$

where *Φ* is the oil volume fraction (0.2), *C* is the CFK concentration in the final emulsion (0.008 g/mL) and 100 corresponded to the dilution factor and a 1 cm path length.

For the determination of foaming capacity (FC) and foaming stability (FS), 5 mL of CFK solution (10 mg/mL in PBS, pH 7.0) was homogenized at 10,000 rpm for 2 min and then transferred to a 25 mL graduated cylinder. The initial solution volume (V_1_, 5 mL), the total volume immediately after homogenization (V_2_), and the total volume after 30 min (V_30_) were recorded. The FC and FS values were calculated using the following equations:

(10) $$FC\;(\%)=\frac{V_{2}-V_{1}}{V_{1}}\times 100$$

(11) $$FS\;\left(\%\right)=\frac{V_{30}-V_{1}}{V_{2}-V_{1}}\times 100$$

### 3.6. Statistical Analysis

All analyses were performed on samples obtained from three independent ultrasound treatment runs, using separately prepared dispersions of the same combined freeze-dried keratin material. Unless otherwise stated, analyses were performed in at least three independent replicates. The data were subjected to analysis of variance (ANOVA), and differences among treatments were determined using Tukey’s multiple comparison test at a 95% confidence level. All statistical analyses were performed using Minitab 16 software (Minitab Inc., State College, PA, USA).

## 4. Conclusions

This study showed that probe ultrasound treatment (30–60% amplitude, 10 min) changed the secondary structure, free –SH content, and techno-functional properties of chicken feather keratin in an amplitude-dependent manner, without marked changes in its characteristic amide bands or apparent molecular-weight profile. FTIR and SDS–PAGE analyses showed that the major amide band positions and electrophoretic profiles of keratin remained largely unchanged across all treatments. However, Amide I deconvolution revealed an amplitude-dependent partial unfolding of native β-sheet structures at higher amplitudes (40–60%), accompanied by new bands in the skeletal region of the FTIR spectra and a loss of free –SH groups.

In parallel with these structural changes, ultrasound treatment resulted in amplitude-dependent changes in functional properties. Surface hydrophobicity, oil-holding capacity, emulsifying activity index, and ABTS-based antioxidant activity were highest at 40% amplitude. In contrast, water solubility and emulsion stability reached their highest values at 60% amplitude, while CUPRAC activity decreased at amplitudes of 40% and above. Ultrasound treatment slightly improved foaming capacity, whereas foam stability and in vitro protein digestibility remained mainly unaffected. These findings suggest that no single ultrasound amplitude improved all functional properties. Therefore, the most suitable treatment depends on the intended application area. The 40% amplitude was more favorable for oil-holding, emulsion formation, and ABTS radical scavenging, whereas 60% amplitude was more favorable for solubility and emulsion stability.

In conclusion, these results suggest that ultrasound treatment can be considered a simple, physical strategy for modifying the techno-functional performance of feather keratin, a by-product of poultry processing. The improved solubility, emulsifying, foaming, and ABTS-based antioxidant properties observed may be of interest for food and personal-care applications. However, this relevance is still theoretical, because only the keratin dispersions were characterized and no formulation, stability, safety, cytocompatibility, or skin/hair performance tests were carried out. In addition, the mechanisms discussed in this study, such as protein unfolding, reaggregation, and thiol oxidation, are proposed explanations and were not directly demonstrated. The primary structure (peptide-bond cleavage and amino acid modifications) was not analyzed, and techniques such as mass spectrometry would be needed for this purpose. Measurements of particle size, droplet size, and interfacial properties would also help to test these explanations. Further studies are needed to evaluate the performance of ultrasound-treated keratin in real food and cosmetic formulation systems, together with its stability, safety, and cytocompatibility. The salt and residual alkali content of the unwashed keratin should also be determined.

## Figures and Tables

**Table 1:** Crude protein content, extraction yield, and protein recovery of chicken feather keratin (CFK).

Properties	Value (%)
Total crude protein content of chicken feathers	87.06 ± 1.24
Total crude protein content of CFK	74.38 ± 0.62
Extraction yield	16.87 ± 0.21
Protein recovery	14.35 ± 0.49

**Table 2:** Relative percentages (%) of secondary structure components of untreated and ultrasound-treated (US30–US60) chicken feather keratin (CFK) samples obtained from FTIR Amide I band deconvolution.

Secondary Structures	Control	US30	US40	US50	US60
β-sheet	28.52 ^a^ ± 0.6	27.66 ^ab^ ± 0.7	27.05 ^bc^ ± 0.4	25.42 ^d^ ± 0.8	26.27 ^cd^ ± 0.5
α-helix	55.54 ± 0.7	55.95 ± 0.9	56.48 ± 0.9	57.16 ± 0.6	56.29 ± 1.1
β-turn	11.39 ± 0.2	11.20 ± 1.1	11.30 ± 0.4	11.80 ± 0.2	12.13 ± 0.5
Intermolecular β-sheet	4.54 ± 0.2	5.12 ± 0.3	5.17 ± 0.4	5.58 ± 0.1	6.02 ± 0.3

Values are expressed as mean ± standard deviation. Different lowercase superscript letters within the same row indicate significant differences among samples (*p* < 0.05). Control represents the untreated chicken feather keratin (CFK) sample, while US30, US40, US50, and US60 represent CFK samples treated with ultrasound at amplitudes of 30, 40, 50, and 60%, respectively.

**Table 3:** ζ-Potential at different pH values and surface hydrophobicity (S_0_) of control (untreated) and ultrasound-treated chicken feather keratin samples.

Sample	ζ-Potential (mV)				Surface Hydrophobicity (S_0_)
	pH 3.0	pH 5.0	pH 7.0	pH 9.0	
Control	34.77 ± 3.84 ^a^	−14.50 ± 0.51	−36.30 ± 3.85	−33.53 ± 2.74	2.19 × 10^4^ ± 0.14 × 10^3^ ^b^
US30	32.17 ± 3.52 ^ab^	−14.70 ± 1.61	−29.87 ± 2.08	−33.73 ± 1.56	2.13 × 10^4^ ± 1.39 × 10^3^ ^b^
US40	27.33 ± 1.03 ^ab^	−14.10 ± 1.61	−32.23 ± 2.82	−34.57 ± 2.45	3.78 × 10^4^ ± 2.23 × 10^3^ ^a^
US50	30.57 ± 1.15 ^ab^	−15.57 ± 0.81	−28.00 ± 2.10	−34.65 ± 2.05	2.47 × 10^4^ ± 0.92 × 10^3^ ^b^
US60	25.33 ± 1.59 ^b^	−16.90 ± 1.77	−30.90 ± 1.34	−32.90 ± 1.74	2.52 × 10^4^ ± 2.66 × 10^3^ ^b^

Values are expressed as mean ± standard deviation. Different lowercase superscript letters within the same column indicate significant differences among samples (*p* < 0.05). Control represents the untreated chicken feather keratin (CFK) sample, while US30, US40, US50, and US60 represent CFK samples treated with ultrasound at amplitudes of 30, 40, 50, and 60%, respectively.

## Data Availability

The raw data supporting the conclusions of this article will be made available by the authors on request.

## Supplementary Materials

The following supporting information can be downloaded at: https://www.mdpi.com/article/10.3390/molecules31214037/s1, Figure S1: Curve fitting and secondary structure deconvolution of the FTIR Amide I band (1600–1700 cm^−1^) of untreated chicken feather keratin; Figure S2: Curve fitting and secondary structure deconvolution of the FTIR Amide I band (1600–1700 cm^−1^) of US30-treated keratin; Figure S3: Curve fitting and secondary structure deconvolution of the FTIR Amide I band (1600–1700 cm^−1^) of US40-treated keratin; Figure S4: Curve fitting and secondary structure deconvolution of the FTIR Amide I band (1600–1700 cm^−1^) of US50-treated keratin; Figure S5: Curve fitting and secondary structure deconvolution of the FTIR Amide I band (1600–1700 cm^−1^) of US60-treated keratin.

## Abbreviations

The following abbreviations are used in this manuscript:

ABTS	2,2′-azino-bis(3-ethylbenzothiazoline-6-sulfonic acid)
ANOVA	Analysis of variance
ANS	8-anilino-1-naphthalenesulfonic acid
CFK	Chicken feather keratin
CUPRAC	Cupric reducing antioxidant capacity
DPPH	2,2-diphenyl-1-picrylhydrazyl
EAI	Emulsifying activity index
ESI	Emulsion stability index
FC	Foaming capacity
FS	Foam stability
FTIR	Fourier Transform Infrared
INCI	International Nomenclature Cosmetic Ingredient
IVPD	In vitro protein digestibility
OHC	Oil-holding capacity
SDS–PAGE	Sodium dodecyl sulfate–polyacrylamide gel electrophoresis
S_0_	Surface hydrophobicity
US	Ultrasound
