# Supplementary material for: Effect of Ultrasound Treatment on the Structural and Techno-Functional Properties of Feather-Derived Keratin
Source: Molecules. 2026 Nov 9;31(21):4037. doi: 10.3390/molecules31214037 (PMC13765403; doi:10.3390/molecules31214037)
Supplement: Supplementary file 1 [file molecules-31-04037-s001.zip › molecules-4589719-supplementary.pdf]

Article

Effect of Ultrasound Treatment on the Structural and Techno-Functional Properties of Feather-Derived Keratin

Nisa Akkuzu [1], Betül Unur [1], Rukiye Gundogan [1], Tuncay Özdemir [2], Gulsah Karabulut [3], Ebru Firatligil [1], and Asli Can Karaca [1,*]

[1] Department of Food Engineering, Faculty of Chemical and Metallurgical Engineering, Istanbul Technical University, 34469 Maslak, Istanbul, Turkiye
[2] Senpilic Gida San. A.S., 54700 Geyve, Sakarya, Turkiye
[3] Department of Food Engineering, Faculty of Engineering, Sakarya University, 54187 Serdivan, Sakarya, Turkiye
* Correspondence: cankaraca@itu.edu.tr

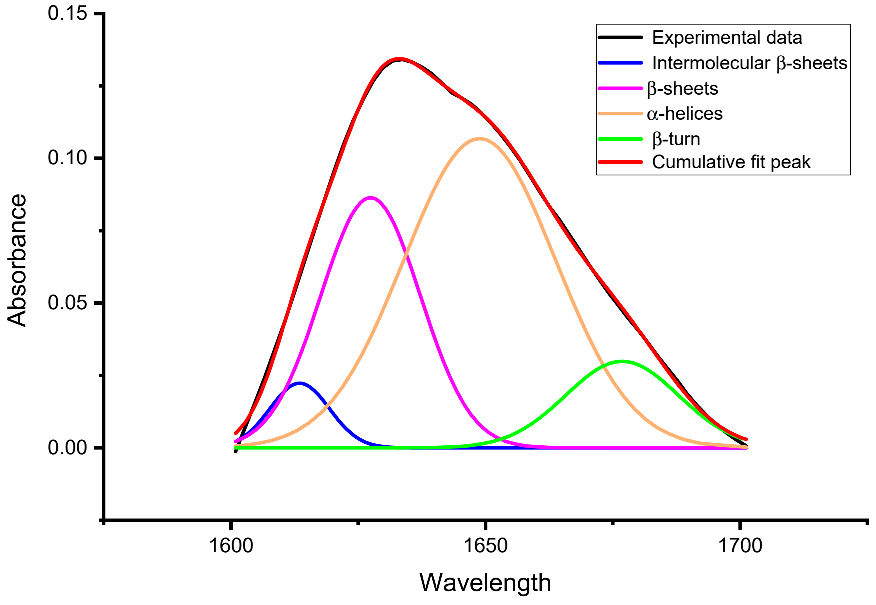

Figure S1. Curve fitting and secondary structure deconvolution of the FTIR Amide I band (1600–1700 cm^{-1}) of keratin.

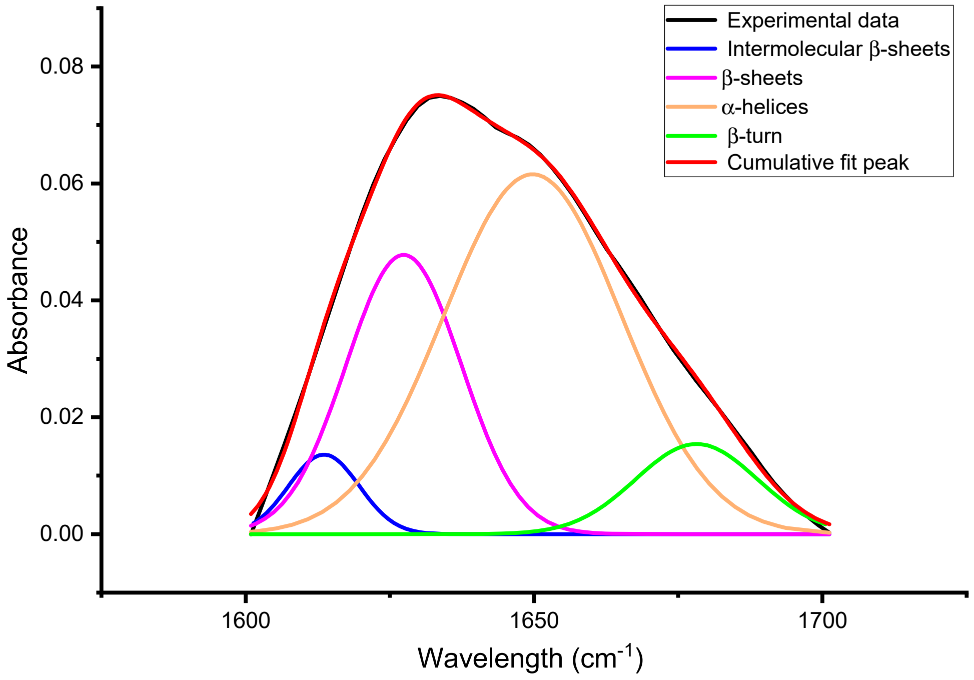

Figure S2. Curve fitting and secondary structure deconvolution of the FTIR Amide I band (1600–1700 cm^{-1}) of US (30W)-treated keratin.

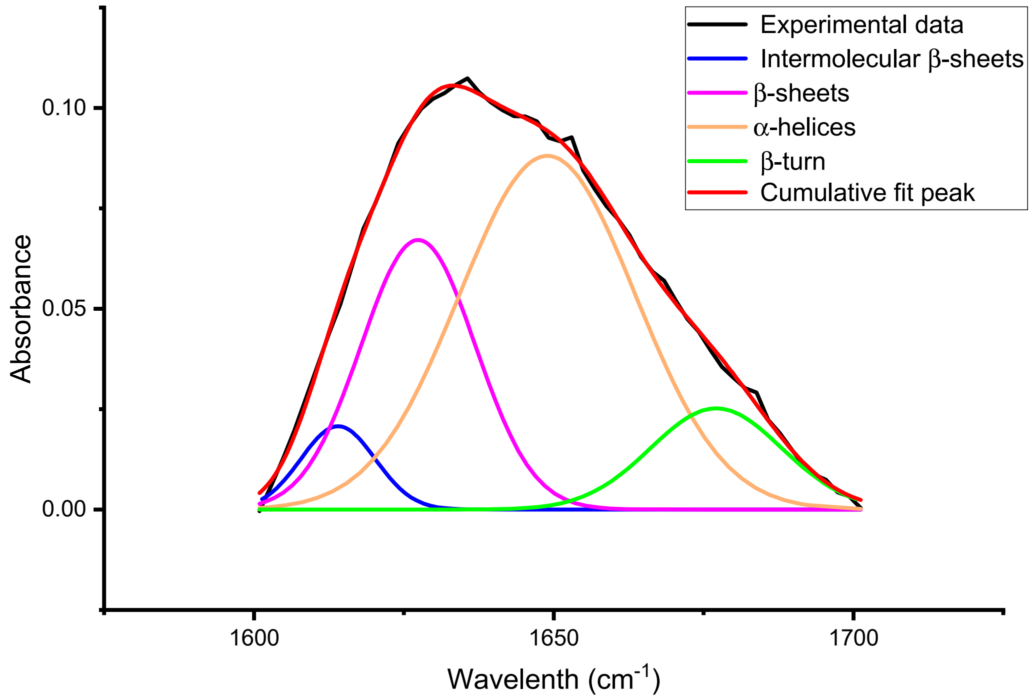

Figure S3. Curve fitting and secondary structure deconvolution of the FTIR Amide I band (1600–1700 cm^{-1}) of US (40W)-treated keratin.

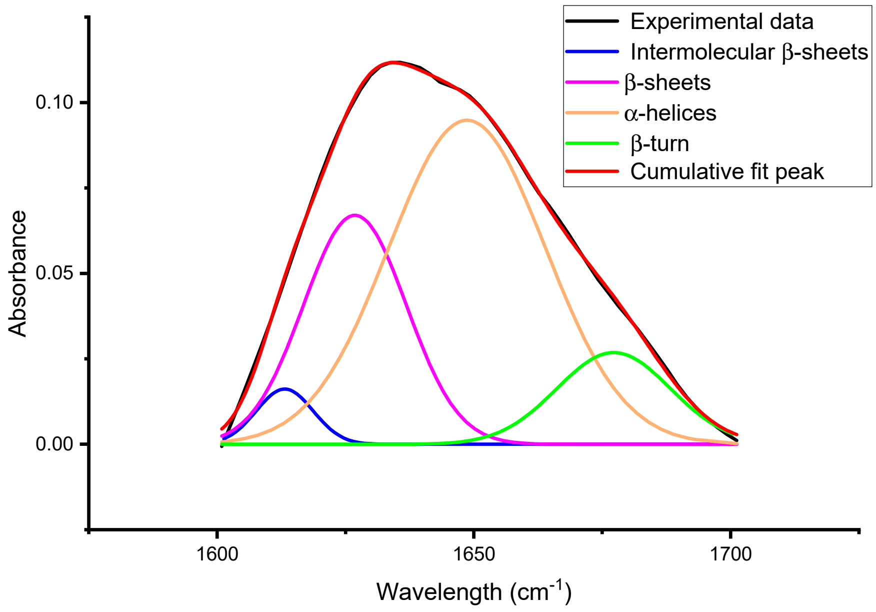

Figure S4. Curve fitting and secondary structure deconvolution of the FTIR Amide I band (1600–1700 cm^{-1}) of US (50W)-treated keratin.

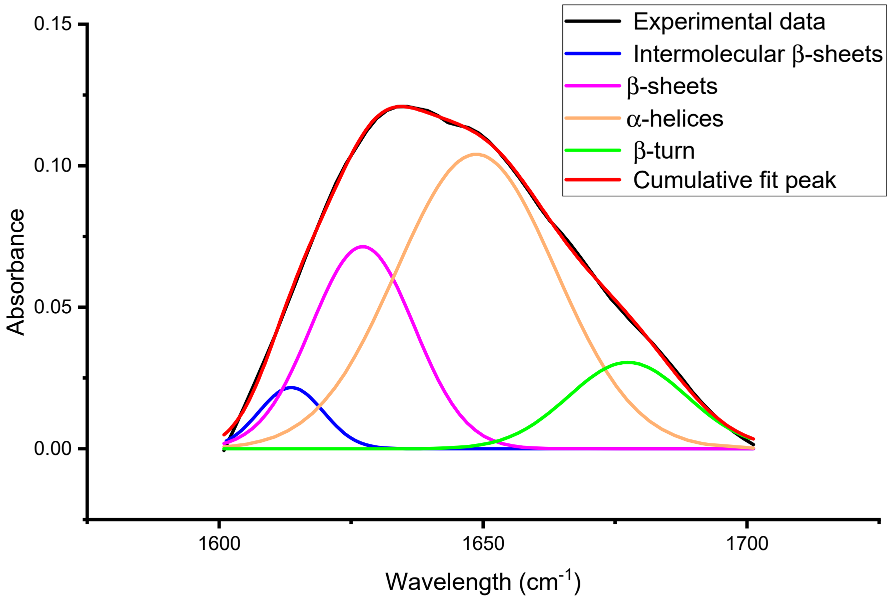

Figure S5. Curve fitting and secondary structure deconvolution of the FTIR Amide I band (1600–1700 cm^{-1}) of US (60W)-treated keratin.
